# Health Literacy and Health Outcomes in Chronic Obstructive Pulmonary Disease Patients: An Explorative Study

**DOI:** 10.3389/fpubh.2022.846768

**Published:** 2022-03-17

**Authors:** Deniz Azkan Ture, Sudip Bhattacharya, Hakan Demirci, Tekin Yildiz

**Affiliations:** ^1^Department of Family Medicine, Bursa Yuksek Ihtisas Training and Research Hospital, University of Health Sciences Turkey, Bursa, Turkey; ^2^Department of Community Medicine, Himalayan Institute of Medical Sciences, Swami Rama Himalayan University, Dehradun, India; ^3^Department of Chest Diseases, Süreyyapaşa Chest Diseases and Thoracic Surgery Training and Research Hospital, University of Health Sciences Turkey, Istanbul, Turkey

**Keywords:** disease severity, GOLD classification, health literacy, HLS-EU-Q, COPD

## Abstract

**Aim:**

This study aimed to evaluate the relationship between health literacy (HL) and chronic obstructive pulmonary disease (COPD) severity.

**Methods:**

Pulmonary function test, sociodemographic features, Modified Medical Research Council (mMRC) dyspnea scale, COPD assessment test (CAT), and the European Health Literacy Survey Questionnaire were used. The study examined 13,760 patients who underwent a pulmonary function test. Out of 13,760 patients, 673 patients had FEV1/FVC values less than 70%. Those with FEV1/FVC< 0.70 (*n* = 336) after the reversibility test were included in the study.

**Results:**

There was a significant decrease in HL and an increase in COPD severity (*p* < 0.001). In multivariate analysis, the risk of severe COPD was 2.74 times higher in patients in the poor income level than in patients in the good income level. In patients with inadequate HL, the risk of developing severe COPD was 1.80 times higher. A significant difference was found in HL index scores among the groups in terms of education level and income level (*p* < 0.001; *p* < 0.001, respectively). The most difficult topics for patients with COPD were periodic health examinations, good practices in mental health, and adult vaccinations.

**Conclusions:**

Patients with COPD were found to be at a HL level well below the expected level. The risk of severe COPD increased with poor income and inadequate HL. Healthcare providers should be careful in accessing, understanding, and interpreting the health information of patients with inadequate HL. Therefore, patient education should be prioritized in the follow-up and in the treatment of patients with COPD. Physicians should pay maximum attention to patients with COPD in the regular use of drugs, their proper use, in taking preventive measures, and in adult vaccination.

## Introduction

The Global Initiative for Chronic Obstructive Lung Disease (GOLD) defines chronic obstructive pulmonary disease (COPD) as follows: “COPD is a common, preventable, and treatable disease that is characterized by persistent respiratory symptoms and airflow limitation that is due to airway and/or alveolar abnormalities usually caused by significant exposure to noxious particles or gases” (1). GOLD suggests the ABCD classification (A: few symptoms, better lung function; B: more symptoms, better lung function; C: few symptoms, poor lung function; D: more symptoms, poor lung function) of the disease using a combined assessment based on an individual's symptoms and history of exacerbations. The prevalence of COPD was 5.9% in the USA (2), 4.56% in the UK, and 12.4%in a European survey (3). COPD is also the fourth leading cause of mortality worldwide (4). In addition, it is one of the leading causes of disability-adjusted life years when disease control cannot be achieved (5).

Chronic obstructive pulmonary disease is a preventable disease. The key point of prevention, promotion, and treatment in COPD is good communication (6). Health literacy (HL) is one of the most important factors that improve communication between healthcare providers and patients in health systems (7). HL is the degree of ability to find, understand, and use health information and healthcare services, and to promote health and prevent disease. Although there are many studies on the effects of HL on chronic diseases (such as diabetes, hypertension, and heart failure), there are very few studies around the world of its effects on COPD (8, 9).

It was reported that there was a decrease in the number of patients with COPD applying for medical care in hospitals following a complex HL intervention; a 39.8% decrease in hospitalization for COPD exacerbations and a 41% decrease in emergency department applications (9).

This study aimed to investigate the relationship between HL and disease severity in patients with COPD.

## Methods

**Figure 1:**
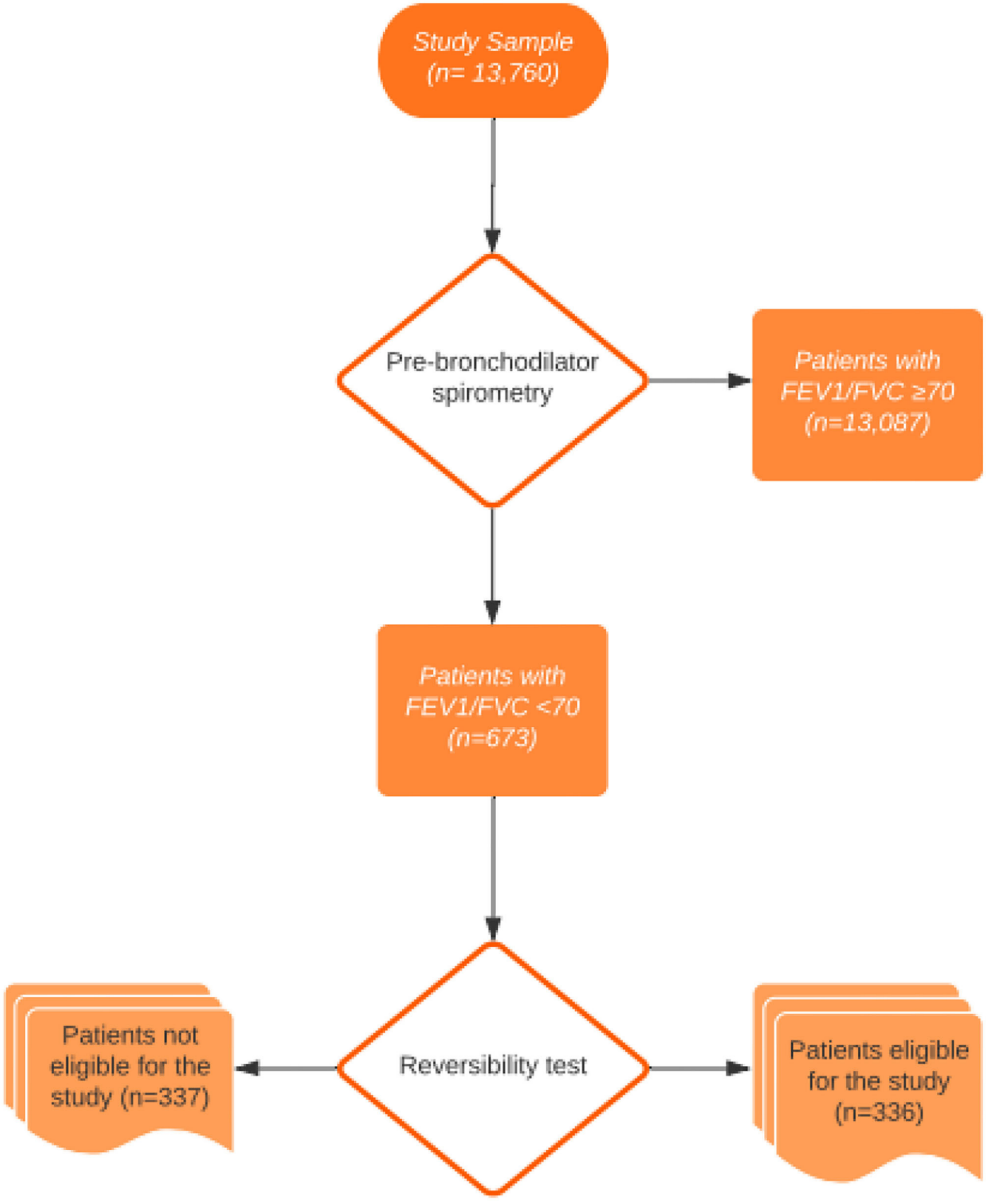
Flow diagram of the participants.

In this study, a total of 13,760 patients admitted to the outpatient clinic of Chest Diseases between February and August 2017 were screened with pulmonary function tests. Of these 13,760 patients, 673 patients had FEV1/FVC values less than 70%. Those with FEV1/FVC < 0.70 were included in the study. That is, if FEV1/FVC was > or = 0.70, the patient was excluded. The study was completed with 336 volunteers from among 673 patients who fulfilled the study criteria. The flow diagram of the participants is shown in Figure 1. Participants were interviewed face-to-face in a comfortable environment where confidentiality of the interview was ensured, and questionnaire forms were filled out. The study was approved by the Bursa Yuksek Ihtisas Training and Research Hospital's Ethics Committee, and written informed consent was obtained from all participants.

Inclusion criteria were COPD subjects over 40 years of age who had <70% FEV1/FVC. Exclusion criteria were pregnancy, being under 40 years old, being subjected to conditions that might have affected cognition (such as hearing or visual problems, history of dementia), FEV1/FVC values more than 70% in the reversibility test, and any disease that might have affected airway problems (any type of respiratory tract cancers).

The COPD assessment test (CAT) (10) and Modified Medical Research Council **(**mMRC) dyspnea score (11) were applied to the participants whose FEV1/FVC was below 70% according to the pulmonary function test to calculate the GOLD score. The reversibility test was performed with 400 mcg salbutamol inhalation to confirm the diagnosis of COPD in patients with FEV1/FVC values < 70% and no previous history of COPD (1).

In the GOLD A, B, C, D classification, spirometry measurement, annual number of exacerbations, number of annual hospitalizations, and symptoms were evaluated together. Cases with a history of at most 1 exacerbation, without hospitalization, and with an mMRC score of 0–1 and a CAT score of <10 were considered as COPD A stage. Cases with a history of at most 1 exacerbation, without hospitalization, and with an mMRC score of ≥2 and a CAT score of ≥10 were COPD B stage. Cases with ≥2 exacerbations and ≥1 hospitalization, as well as with an mMRC score of 0–1 and a CAT score of <10 were COPD C stage. Cases with ≥2 exacerbations and ≥1 hospitalization, as well as with an mMRC score of ≥2 and a CAT score of ≥10 were considered COPD D stage (1). In the GOLD 1, 2, 3, 4 classification, patients are evaluated according to spirometry results. Spirometry results of patients with 80%≤FEV1 are considered as GOLD Stage 1, 50%≤FEV1<80% as GOLD Stage 2, 30%≤FEV1<50% as GOLD Stage 3, and FEV1<30% as GOLD Stage 4 (1).

Health literacy was evaluated with the Turkish translation of the Health Literacy Survey-European Union (HLS-EU) Scale (12, 13). The HLS-EU contains 47 questions, each of which is evaluated between 1 and 4. The validity and reliability of the scale were established by Abacigil et al. (14). According to the original methodology, at least 80% of the questions need to be answered to use a question in index calculations. In the evaluation of HLS-EU, questions 1–47 indicate the general HL index, questions 1–16 indicate the health care HL subindex, questions 17–31 indicate the disease prevention HL subindex, and questions 32–47 indicate the health promotion HL subindex. Index points were calculated, and the general HL was divided into four groups. These groups were “inadequate” (0–25), “limited” (>25–33), “adequate” (>33–42), and “excellent” (>42–50) HL.

### Statistical Analysis

The consistency of continuous variables with normal distribution was examined with the Shapiro–Wilk test. Variables were defined by mean, standard deviation, and median (minimum: maximum) values. Depending on the test variable, comparisons between two groups were made by the *t*-test, Mann–Whitney test, or Chi-square test. Spearman rank correlation coefficient was used to investigate the relationship between two variables. Logistic regression analysis was performed to determine the risk factors associated with severe COPD level. Statistical analyses were performed using SPSS version 23.0 (SPSS Inc., Chicago, IL, USA). A *p-*value of < 0.05 was considered statistically significant for all statistical comparisons.

## Results

The mean age of the participants was 62.53 ± 10.04 years. Of the patients included in the study, 14.58% were women (*n* = 49) and 85.42% were men (*n* = 287).

**Figure 2:**
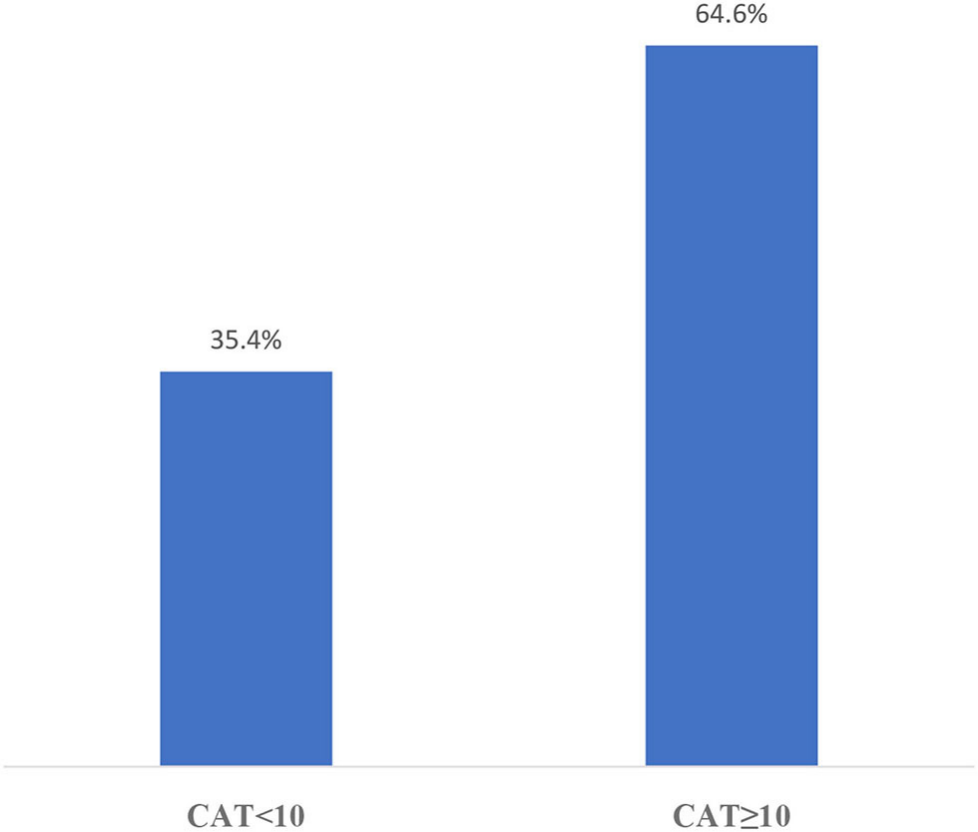
Distribution of COPD assessment test (CAT) results.

**Figure 3:**
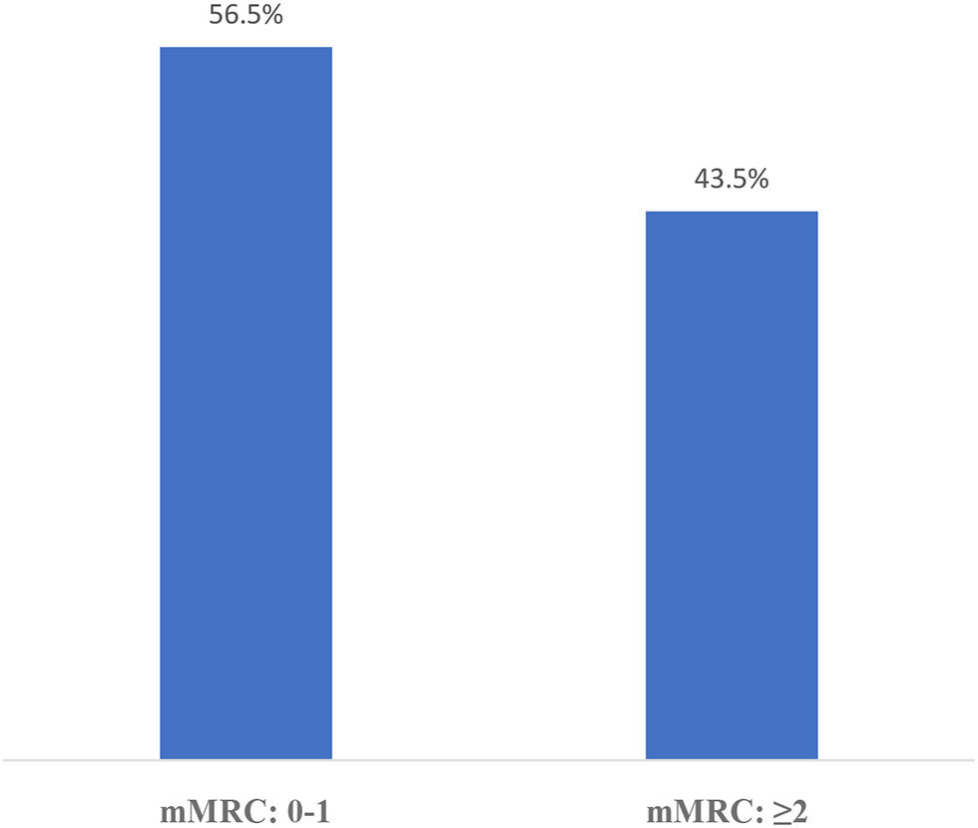
Distribution of mMRC of the participants.

**Figure 4:**
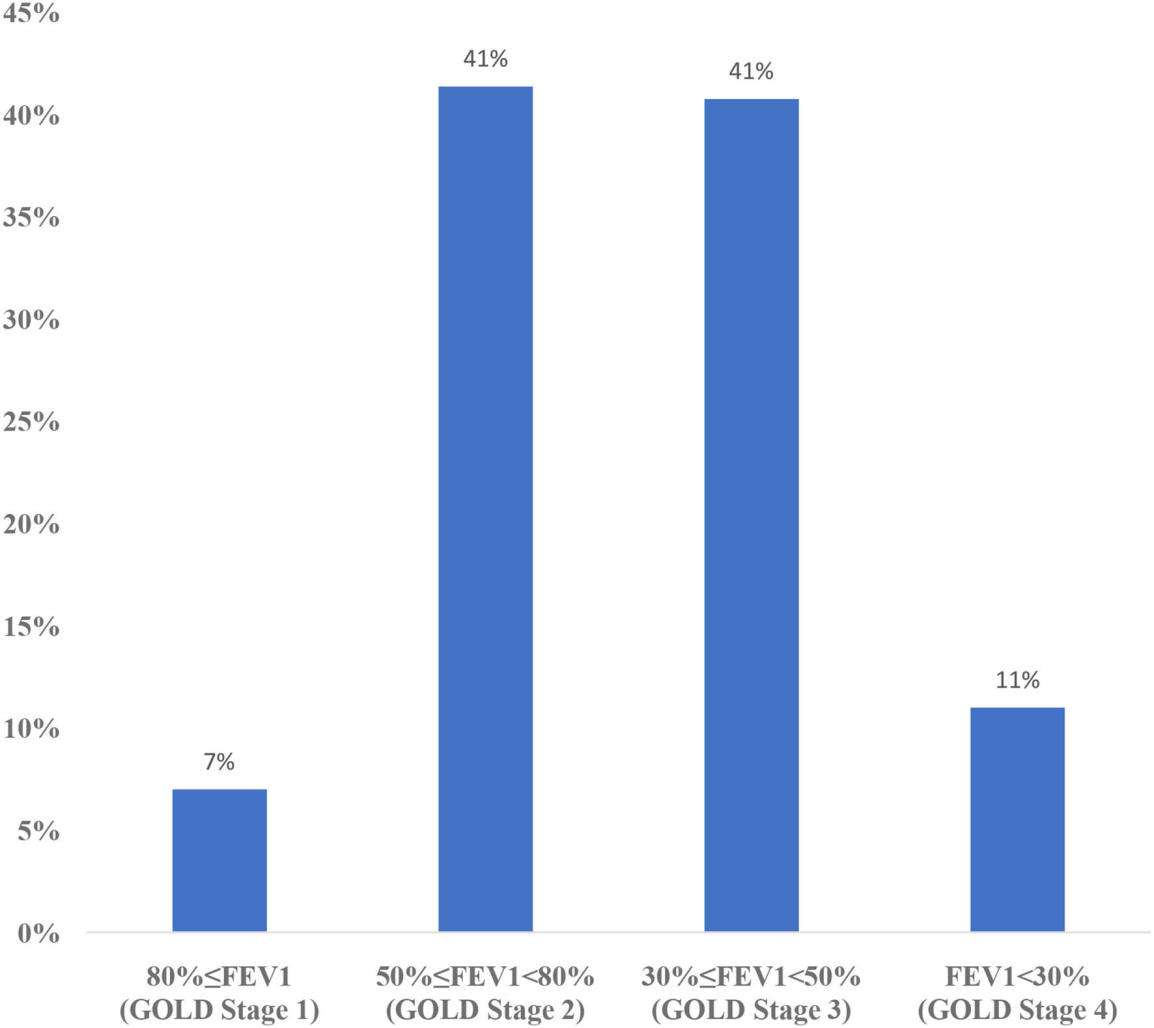
Distribution of GOLD stages (1, 2, 3, 4) according to FEV1 of the participants.

**Figure 5:**
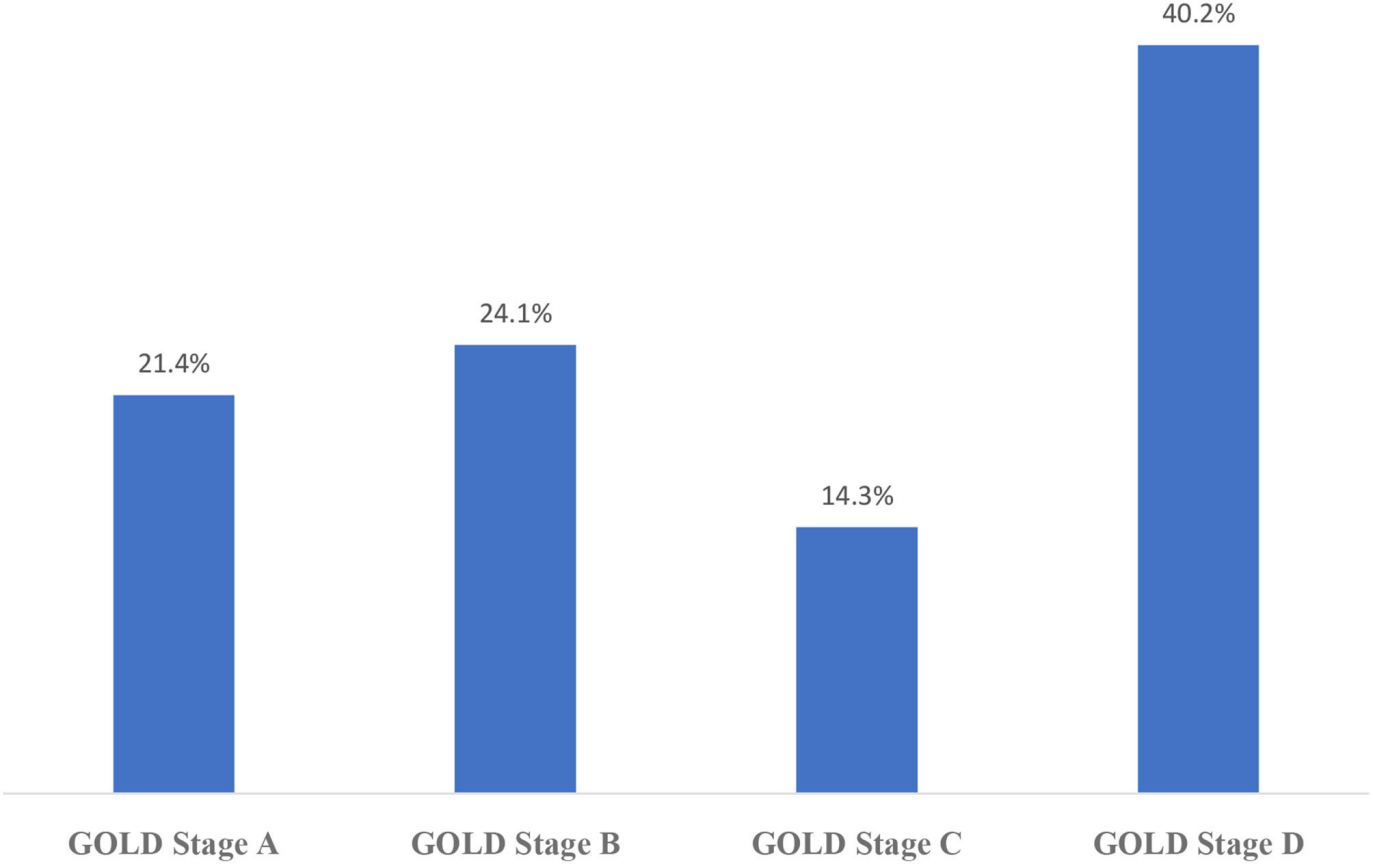
Distribution of GOLD stages (A, B, C, D) of the participants.

The distribution of CAT results in this sampling is shown in Figure 2 and mMRC results are shown in Figure 3. According to the GOLD staging system based on spirometry only, the distribution of patients was 11.3% in Stage 1, 41.4% in Stage 2, 40.8% in Stage 3, and 6.5% in Stage 4 (Figure 4). However, according to GOLD 2021, which includes symptomatology, 21.4% of our patients were in Group A, 24.1% in Group B, 14.3% in Group C, and 40.2% in Group D (Figure 5).

The general characteristics of the participants and their HL levels are shown in Table 1. A significant difference was found in HL index scores among the groups in terms of education level and income level (*p* < 0.001; *p* < 0.001, respectively). There was no difference among the groups in age, gender, or marital status.

**Table 1:** General characteristics of the participants and their health literacy levels.

	**Inadequate health literacy (*n =* 217)**	**Limited/adequate/excellent health literacy (*n =* 119)**	*p*-value (*significant at < 0.05 level)
Age	63.26 ± 10.29 (40:87)	61.22 ± 9.50 (40:84)	0.075
Sex (F/M)	190 (87.60%)/27 (12.40%)	97 (81.50%)/22 (18.50%)	0.133
**Education level**			
Primary (≤ 8 years)	210 (96.8%)	79 (66.40%)	<0.001
Secondary (9-12 years)	6 (2.80%)	26 (21.80%)	
Tertiary (≥ 12 years)	1 (0.50%)	14 (11.80%)	
**Marital status**			
Married	117 (81.60%)	99 (83.20%)	0.710
Single	40 (18.40%)	20 (16.80%)	
**Income**			
Poor	102 (47.01%)	23 (19.33%)	<0.001
Moderate	87 (40.09%)	63 (52.94%)	
Good	28 (12.90%)	33 (27.73%)	

*Data expressed as mean ± standard deviation (minimum: maximum) and n (%)*.

It was determined that 64.6% of the population was in the category of inadequate HL (Table 2). The subjects where patients felt themselves most inadequate were health screening (85.4% inadequate), good practices in mental health (82.4% inadequate), vaccinations (81.5% inadequate), being a member of a sports clubs (81% inadequate), and going to a doctor for check-up purposes for decision making (80.7% inadequate).

**Table 2:** Distribution of health literacy levels of the participants.

	**Inadequate (%)**	**Limited (%)**	**Adequate (%)**	**Excellent (%)**
General health literacy	64.60	28.90	6	0.60
Health care				
health literacy	48.20	33.60	15.50	2.70
Disease prevention health literacy	70.80	20.60	8	0.60
Health promotion health literacy	69	24.70	5.70	0.60

The variables associated with severe COPD are given in Table 3. There was no difference between mild and severe COPD groups according to age and gender (*p* = 0.517 and *p* = 0.684), but it was determined that there was a difference between the groups according to smoking status (*p* = 0.038). In the subgroup analyses, the proportion of patients using active cigarettes was higher in the mild COPD group (51.60 vs. 38.30%; *p* < 0.05), whereas the ex-smoker ratio was higher in the severe COPD group (39.20 vs. 52.50%; *p* < 0.05). There was no difference between the groups according to BMI level (*p* = 0.198). It was determined that there was no difference between the groups according to the level of education (*p* = 0.099). It was seen that there were differences between the groups according to occupation, and it has been determined that the proportion of occupied patients was higher in the mild COPD group (90.80 vs. 81.40%; *p* = 0.014). The income status also differed between the groups (*p* < 0.001). In the subgroup analyses, the proportion of patients with poor income was found to be higher in the severe COPD group (23.50 vs. 48.60%). There was also a difference between the groups according to the level of HL, and it was determined that the proportion of patients with inadequate HL was higher in the severe COPD group (24.20 vs. 73.20%).

**Table 3:** General characteristics of participants and COPD severity.

	***n***	**Mild-moderate COPD**	***n***	**Severe-very severe COPD**	*p*-value (*significant at < 0.05)
**Age (years)**	153	63 (40:87)	183	63 (42:84)	0.517
**Sex (Female/Male)**	153	21/132	183	28/155	0.684
**Tobacco smoke**					
Active		79 (51.60%)		90 (38.30%)	
Ex-smoker	153	60 (39.20%)	183	96 (52.50%)	0.038
Never smoked		14 (9.20%)		17 (9.30%)	
**Body Mass Index (kg/m** ^**2**^ **)**	152	26.12	183	0.198	
		(18.04:50.78)		(13.15:46.61)	
**Education**					
Primary (8 years)		126 (82.40%)		165 (90.20%)	
Secondary (12 years)	153	16 (10.50%)	183	12 (6.60%)	0.099
Tertiary (≥14 years)		11 (7.20%)		6 (3.30%)	
**Occupation**					
Occupied	153	139 (90.80%)	183	149 (81.40%)	0.014
Non-occupied		14 (9.20%)		34 (18.60%)	
**Income**					
Poor		36 (23.50%)		89 (48.60%)	
Moderate	153	81 (52.90%)	183	69 (37.70%)	<0.001
Good		36 (23.50%)		25 (13.70%)	
**Health Literacy**					
Limited/adequate/ excellent	153	70 (45.80%)	183	49 (26.80%)	<0.001
Inadequate		83 (54.20%)		134 (73.20%)	

*Data are expressed as median (minimum: maximum) and n %*.

The results of the analysis of risk factors affecting the degree of COPD at the severe level are given in Table 4. After univariate logistic regression analysis, the variables that met the *p* < 0.25 condition were included in multivariate logistic regression analysis. In the multivariate analysis phase, the backward elimination approach was used as the variable selection method. It was determined that the model obtained at the final step was significant (*p* < 0.001), and it was determined that it was suitable for the data set (*p* = 0.559).

**Table 4:** Factors affecting COPD severity.

	**Univariate analysis**			**Multivariate analysis**		
	**Wald**	**OR (CI95%)**	***p*-value**	**Wald**	**OR (CI95%)**	***p*-value (significant at < 0.05 level)**
**Age (years)**	0.57	1.01 (0.99:1.03)	0.452			
**Sex (Male)**	0.17	0.88 (0.48:1.62)	0.684			
**Tobacco Smoke**						
Active	0.64	0.73 (0.34:1.59)	0.427			
Ex-smoker	0.48	1.32 (0.61:2.87)	0.487			
**Body mass index (kg/m** ^**2**^ **)**	1.33	0.98 (0.94:1.02)	0.248			
**Education**						
Primary (8 years)	2.83	2.40 (0.87:6.67)	0.093			
Secondary (12 years)	0.25	1.38 (0.40:4.78)	0.616			
**Occupation** (non-occupied)	5.83	2.27 (1.17:4.40)	0.016	2.99	1.85 (0.92:3.70)	0.084
**Income**						
Poor	15.10	3.56 (1.88:6.76)	<0.001	8.77	2.74 (1.41:5.38)	0.003
Moderate	0.44	1.23 (0.67:2.24)	0.507	0.14	1.13 (0.61:2.08)	0.707
**Health literacy** (*Inadequate)*	12.88	2.31 (1.46:3.64)	<0.001	5.75	1.80 (1.11:2.92)	0.016

*OR, Odds ratio; CI, Confidence interval. Reference Categories: Sex (female), Tobacco smoke (Never smoked), Tertiary (≥14 years), Occupation (occupied), Income (good), Health literacy (limited/adequate/excellent)*.

When the table is examined, it is seen that the risk of severe COPD is 2.74 times higher in patients in the poor income level than in patients in the good income level. In patients with inadequate HL, the risk of developing severe COPD was 1.80 times higher.

## Discussion

There was a significant decrease in HL with an increase in COPD severity. The risk of severe COPD was higher in patients in the poor income level than in patients in the good income level. In patients with inadequate HL, the risk of developing severe COPD was higher. The rate of primary education was higher in the inadequate HL group. Poor economic status was also higher in the inadequate HL group. The most difficult topics for patients with COPD were periodic health examinations, good practices in mental health, and adult vaccinations.

The relationship between other chronic diseases and HL has been demonstrated in previous studies (15). For example, many studies reported that blood sugar control in patients with DM is better in patients with good HL (16). Similarly, studies have shown that good HL is protective against complications in patients with diabetes mellitus (17). Patients with better HL also have lower insulin consumption among patients with DM (18). Another common chronic disease in the community is hypertension. Similar situations exist for patients with hypertension. Inadequate HL has been shown to increase the morbidity and mortality associated with COPD (9). It has been reported that patients with COPD who had good HL used inhalers less, and the cost of disease decreases with good HL (19). In this study, it was found that COPD severity was higher in patients with poor HL. This can be explained by the high compliance of these patients with doctor's recommendations.

Education level is one of the most important factors affecting HL. As education levels decrease, HL levels also decrease (20). Even if sociodemographic data, including education and income, are improved, COPD is associated with insufficient HL on its own (21). Patients with COPD with low HL have worse test values of lung function capacity compared to patients with adequate lung HL and they experience more difficulties in daily life activities, become more dependent individuals, and experience more multimorbidities, leading to lower survival expectations (8). In the current study, it was found that there was a relationship between education and income level and HL in patients with COPD.

Educating people with COPD about their condition will enable them to acquire healthier behaviors, especially by changing awareness about smoking, which is known to be the most important etiological factor for COPD (1). In our study, we found that 44% of the cases were active smokers and 46% were former smokers. Also 85.1% of our patients understood the impact of smoking, lack of physical activity, and excessive use of alcohol on their own health. Although this value is above the average for Turkey (79%), it is below the European average (89.7%). In a study examining the relationship between HL and COPD by Puente-Maestu et al. it was found that 39.1% of the patients were smokers and 46.1% of these patients had adequate HL (8). Even though the rate of smokers was close to that in our study, the rate of HL for cigarette consumption was high in our findings.

In research studies, the prominent issues related to HL are age, gender, education, and economic status (22–24). In this study, there was no difference between the HL groups in terms of age and gender. This may be since very few participants were identified at the level of adequate and excellent HL. Therefore, in this study, analyses were done by separating HL groups as insufficient or others. We found that the income status was clearly different between HL groups. The inadequacy of HL in individuals with low income can be partially explained by the fact that these individuals also have a lower education level. The ability of individuals with a higher income to seek better treatment for their health could be another important factor.

In the present study, participants had difficulty in deciding which vaccines they needed. GOLD and our national guidelines recommend influenza and pneumococcal (PCV13 and PPSV23) vaccines for patients with COPD (1). Vaccines are provided free of cost in our country. Adult vaccination is not seen as a part of preventive health services because of the doubts about the effectiveness and side effects of vaccines, the non-existence or the lack of national health policy for adult immunization, and the economic burden of vaccines on governments (25). In primary care, family physicians should always remind adult patients to have their children vaccinated and encourage them to receive vaccinations.

Adequate HL increases participation in cancer screening (26). In a study questioning the relationship between HL and health screening in women, it was shown that improving HL should be an integral part of breast and cervical cancer screening programs. In the same study, it was predicted that HL-based intervention studies could increase cancer screening in addition to breast and cervical cancer in different ethnic groups (27). The role of HL also played a crucial role during the COVID-19 pandemic (28–30). In the present study, one of the most challenging questions for patients was “deciding which health screening you need”. About 85.4% of the patients answered this question as “fairly difficult and very difficult.” Patients with inadequate HL should be better informed about adult immunizations.

The present study has been done with some unavoidable limitations. It was a cross-sectional study that included patients who applied to the Chest Diseases Clinic and reflected local characteristics. The number of patients with early-stage disease may be expected to be higher in a larger population-based sample. In addition, HL rates may be different in primary care since it is known that patients with low HL are admitted to hospitals at a higher rate. It can therefore be suggested to carry out new studies in this field at an early stage.

In conclusion, patients with COPD were found to be at an HL level well below the expected level. The risk of severe COPD increased with poor income and inadequate HL. Healthcare providers should be careful in assessing, understanding, and interpreting the health information of patients with inadequate HL. Therefore, patient education should be prioritized in the follow-up and treatment of patients with COPD. Physicians should pay maximum attention to patients with COPD in the regular use of drugs, their proper use, in taking preventive measures, and in adult vaccination.

## Data Availability Statement

The raw data supporting the conclusions of this article will be made available by the authors, without undue reservation.

## Ethics Statement

The study was approved by the Bursa Yuksek Ihtisas Training and Research Hospital's Ethics Committee and written informed consent was obtained from all participants.

## Author Contributions

DAT, HD, and TY conceived the study, produced the first draft, and all authors contributed to interpretation of the findings. SB has given inputs and revised the draft. All authors has approved the final draft.

## Conflict of Interest

The authors declare that the research was conducted in the absence of any commercial or financial relationships that could be construed as a potential conflict of interest.

## Publisher's Note

All claims expressed in this article are solely those of the authors and do not necessarily represent those of their affiliated organizations, or those of the publisher, the editors and the reviewers. Any product that may be evaluated in this article, or claim that may be made by its manufacturer, is not guaranteed or endorsed by the publisher.
